# Infant Nasopharyngeal Microbiota Subphenotypes and Early Childhood Lung Function: Evidence from a Rural Ghanaian Pregnancy Cohort

**DOI:** 10.3390/ijerph18147276

**Published:** 2021-07-07

**Authors:** Kathryn Dubowski, Seyram Kaali, Darby Jack, Rebecca Kyerewaa Dwommoh Prah, Jose C. Clemente, Theresa Tawiah, Mohammed Mujtaba, Louisa Iddrisu, Daniel Carrión, Dennis Gyasi Konadu, Oscar Agyei, Francis Mensah Kornu, Samuel Osei-Owusu, Alison G. Lee, Kwaku Poku Asante

**Affiliations:** 1 Division of Pulmonary, Critical Care and Sleep Medicine, Icahn School of Medicine Mount Sinai, New York, NY 10029, USA; Alison.Lee@mssm.edu; 2 Kintampo Health Research Centre, Kintampo 00233, Ghana; kaali.seyram@kintampo-hrc.org (S.K.); rebecca.dwommoh@gmail.com (R.K.D.P.); theresa.tawiah@kintampo-hrc.org (T.T.); mohammed.mujtaba@kintampo-hrc.org (M.M.); louisa.iddrisu@kintampo-hrc.org (L.I.); dennis.konadu@kintampo-hrc.org (D.G.K.); oscar.agyei@kintampo-hrc.org (O.A.); Francis.Kornu@kintampo-hrc.org (F.M.K.); samuel.osei-owusu@kintampo-hrc.org (S.O.-O.); kwakupoku.asante@kintampo-hrc.org (K.P.A.); 3 Department of Environmental Health Sciences, Columbia University Mailman School of Public Health, New York, NY 10032, USA; dj2183@cumc.columbia.edu; 4 Department of Genetics and Genomic Sciences and Medicine, Icahn School of Medicine Mount Sinai, New York, NY 10029, USA; jose.clemente@mssm.edu; 5 Department of Environmental Health Sciences, Icahn School of Medicine Mount Sinai, New York, NY 10029, USA; daniel.carrion@mssm.edu

**Keywords:** nasopharyngeal microbiota, impulse oscillometry, global health

## Abstract

Early life respiratory microbiota may increase risk for future pulmonary disease. Associations between respiratory microbiota and lung health in children from low- and middle-income countries are not well-described. Leveraging the Ghana Randomized Air Pollution and Health Study (GRAPHS) prospective pregnancy cohort in Kintampo, Ghana, we collected nasopharyngeal swabs in 112 asymptomatic children aged median 4.3 months (interquartile range (IQR) 2.9, 7.1) and analyzed 22 common bacterial and viral pathogens with MassTag polymerase chain reaction (PCR). We prospectively followed the cohort and measured lung function at age four years by impulse oscillometry. First, we employed latent class analysis (LCA) to identify nasopharyngeal microbiota (NPM) subphenotypes. Then, we used linear regression to analyze associations between subphenotype assignment and lung function. LCA suggest that a two-class model best described the infant NPM. We identified a higher diversity subphenotype (N = 38, 34%) with more pathogens (median 4; IQR 3.25, 4.75) and a lower diversity subphenotype (N = 74, 66%) with fewer pathogens (median 1; IQR 1, 2). In multivariable linear regression models, the less diverse NPM subphenotype had higher small airway resistance (R5-R20 β = 17.9%, 95% CI 35.6, 0.23; *p* = 0.047) compared with the more diverse subphenotype. Further studies are required to understand the role of the microbiota in future lung health.

## 1. Introduction

Impairment in early childhood lung development has implications for respiratory morbidity and mortality over the life course. Poor lung function has been associated with respiratory illness, including pneumonia, a leading cause of morbidity and mortality in children worldwide and especially in low- and middle-income countries (LMICs) [1,2,3]. Cohort studies suggest that lower lung function by six years of age or earlier is associated with lower lung function in adulthood [4,5]. Decrements in early life lung function may represent structural changes that persist into adulthood thereby increasing risk for future chronic respiratory disease such as chronic obstructive pulmonary disease [5,6,7,8,9,10,11,12,13]. A critical step in understanding lifelong risk is characterizing mechanisms that lead to and maintain this early predisposition.

Emerging literature, mainly from high-income countries with very limited data from LMICs, is beginning to elucidate the importance of the respiratory microbiome on lung development. The respiratory tract serves as a rich environment for microbial-host interactions. A diverse respiratory microbiome is present within hours of birth and evolves dramatically from birth to age 3 years [14,15,16,17,18]. In high-income countries, repeated upper respiratory tract microbiome analyses suggest that tightly-orchestrated compositional changes in early childhood are influenced by environmental factors, such as tobacco smoke exposure, and individual-level factors, such as mode of delivery and parity [19,20]. The upper airway microbiome of healthy children provides a reasonable surrogate for the lower airway microbiome, supporting methodologies that leverage noninvasive nasal sampling of healthy children to investigate associations with early life lung health [21].

Dysbiosis, or alterations in microbe colonization of the upper airway, has been associated with respiratory disease. Disruptions in microbiome maturation may lead to airway inflammation [22], chronic wheeze and asthma [20,23,24], and pneumonia [15,25,26]. Specifically, *Cornebacteriacae* and *Staphylococcus* species which are earlier colonizers of the respiratory tract, may decrease risk for childhood wheeze and asthma [16]. *Streptococcus*, *Haemophilus*, and *Moraxella* species tend to colonize the respiratory tract later in development. However, when they appear earlier, they are often associated with increased risk of wheeze and the development of asthma [19,20,24]. Data from our cohort in rural Ghana suggests a possible link between early life colonization with bacterial pathogens, specifically *Streptococcus pneumonia, Haemophilus influenza, Moraxella cattarhalis*, and infant pneumonia [27]. It is unknown whether pathogen colonization is linked to other objective measures of future lung health, such as lung function.

The overall microbiome diversity may correlate with lung function and subsequent respiratory illness. Loss of diversity has been associated with increased incidence of disease and disease severity in multiple respiratory illnesses [28]. For example, loss of nasal microbiome diversity was associated with increased risk for rhinitis in infants in Singapore and asthma in children from five rural areas in Europe [16,29]. Animal studies support these associations and suggest a role of early life respiratory microbiome on lung development. For example, germ-free mice have fewer alveoli and smaller lungs; colonization with *Lactobacillus* spp. normalizes alveolar numbers [30,31]. Ovalbumin sensitized germ-free mice demonstrate increased airway resistance; reconstitution of microbiota at birth reverses these findings. As microbes do not exist in isolation, an understanding of subphenotypes of pathogens may be necessary to understand how the respiratory microbiome shapes lung development.

Multiple techniques for investigating the microbiome have been presented in the literature, including 16S ribosomal RNA gene sequencing and shotgun metagenomics gene sequencing, and whole-genome sequencing [32,33]. These methods allow for culture independent examination of the microbiome with varying levels of specificity. Alternatively, polymerase chain reaction (PCR), a culture independent technique which detects pre-specified organisms, providing a sample of the microbiota of the lung, but not a comprehensive assessment of the microbiome [34]. This technique has also been used in the literature to explore respiratory microbiota [21].

To our knowledge, the role of respiratory microbiota subphenotypes on lung function has not been fully elucidated in a rural, sub-Saharan population. We therefore leveraged a pregnancy cohort from Kintampo, Ghana, to examine the relationship between infant nasopharyngeal bacterial and viral carriage as defined by PCR and early childhood lung function. Specifically, we employed latent class analysis to determine subphenotypes of bacterial and viral carriage in infancy. We then used linear regression to understand associations between the identified subphenotypes and lung function at child age four years as measured by impulse oscillometry (IOS).

## 2. Materials and Methods

### 2.1. Study Participants

Mother–infant dyads were recruited from a pregnancy cohort derived from the Ghana Randomized Air Pollution and Health Study (GRAPHS) with ongoing, prospective follow up through child age four years. The GRAPHS cohort has been described elsewhere [35]. Briefly, pregnant nonsmoking women were recruited prior to ultrasound-confirmed 24 weeks of gestation. Key individual and household level data were collected on enrollment, including maternal age, parity, ethnicity, and number of people living in the household. Fieldworkers trained in the World Health Organization’s Integrated Management of Childhood Illness (IMCI) performed weekly surveillance of study children over the first year of life and referred any child meeting criteria for pneumonia or who was otherwise unwell for physician evaluation and pneumonia diagnosis. Nasopharyngeal microbiota (NPM) swabs were collected for children with physician-diagnosed pneumonia and age- and sex-matched healthy controls (total N = 669). Children are being followed in ongoing prospective work with respiratory phenotyping, including lung function measured by IOS at child age four years. The current analyses include a convenience sample of N = 112 children without respiratory symptoms at the time of nasopharyngeal swab and who successfully performed IOS at child age four years.

### 2.2. Nasopharyngeal Microbiota Sampling and PCR

As above, NPM swabs were collected in infants with and without respiratory symptoms. These analyses include only children with no respiratory symptoms at time of swab and who did not have a physician-diagnosed pneumonia episode within two weeks prior to or following swab collection. As previously described, swabs were stored at −80 °C until analysis; all samples were shipped from Ghana to the United States on dry ice with internal temperature monitors. We employed MassTag polymerase chain reaction (PCR) to identify the presence of 22 common bacterial and viral respiratory pathogens (see Table S1) [27]. We extracted total nucleic acid (TNA) from 200 mL of each nasal swab using the EasyMag Extraction Platform (Biomerieux, Marcy l’Etoile, France). TNA was eluted in 40 mL. Ten mL of TNA DNA was used to generate cDNA. TNA or cDNA (5 mL per reaction) was then used as a template for MassTag PCR using two separate PCR multiplex assays. The first PCR multiplex assay targeted common respiratory RNA viruses with cDNA as the template. The second targeted bacterial agents and adenovirus using TNA as the template. PCR and agent detection were performed as previously described [36].

### 2.3. Impulse Oscillometry (IOS) Lung Function Testing

Lung function was measured in the children at four years of age by a trained study pediatrician using impulse oscillometry (IOS) (Masterscreen IOS, Carefusion, San Diego, CA, USA) following American Thoracic Society guidelines [37,38,39]. Eligible participants were free from respiratory symptoms for at least three weeks. IOS requires that a participant perform tidal breathing into a mouthpiece for 30 seconds and therefore, contrary to spirometry, is not reliant on participant effort and can be reliably performed in young children. The IOS system was calibrated each day immediately prior to measurements using a standard 3-liter syringe. The participants performed IOS in a standing position and wearing a nose clip with cheeks supported by a trained research assistant. The supervising pediatrician evaluated efforts and made sure that each valid study included at least three reproducible measures free from artifact. The Masterscreen LabManager calculated R_rs_ and X_rs_ and reported resistance at 5 Hz (R5), reactance at 5 Hz (X5), resistance at 20 Hz (R20), the difference in resistance at 5 and 20 Hz (R5-20), reactance area (AX), and resonant frequency (Fres). Mean values from the three valid trials were used in analyses.

### 2.4. Covariates

Maternal education, parity, secondhand smoke exposure (defined as having a household member who smoked), ethnicity, and number of persons living in the household were the categorical variables obtained via questionnaire at study enrollment. Child sex and date of birth were recorded at delivery. Age at time of NPM swab was determined by the difference between date of NPM swab and date of delivery. Child height and weight were measured at the time of lung function testing.

### 2.5. Statistical Analysis

First, we performed a latent class analysis of the NPM PCR data to identify the number of subphenotypes of pathogens, considering two to five classes. Criteria for model selection, where the number of subphenotypes defines the model, included Akaike information criterion (AIC) and Bayesian information criterion (BIC), where smaller values suggest better fit; entropy, range 0 to 1 where higher values indicate better class separation; and subphenotype class size, where classes with small numbers would not be beneficial [40]. Once the optimal number of subphenotypes was identified, participants were assigned to the subphenotype for which they had the highest probability of correct assignment. These NPM subphenotype assignments were then used as the independent variable for subsequent regression analyses.

We employed bivariate and multivariable linear regression to determine associations between the identified subphenotypes and IOS variables. Secondary analyses included exploring whether specific pathogens drove any identified associations with IOS variables. Multivariable models were adjusted for child sex, height, age at NPM swab, time between NPM swab and lung function, and maternal education. Sensitivity analyses additionally adjusted for parity, secondhand smoke exposure, number of people living in the household, race/ethnicity, and child BMI.

### 2.6. Internal Review Board Approval

IRB approval was obtained from the Icahn School of Medicine Mount Sinai, Columbia University, and Ghana Interval Review Boards.

## 3. Results

Of the 112 children included in the study, *n* = 57 (50.9%) were male (Table 1). Children were a median age of 4.27 (IQR 2.86, 7.11) months at the time of NPM swab with a median time of 3.57 (IQR 3.37, 3.70) years between swab and lung function. Only 11 (9.8%) mothers reported secondhand smoke exposure. Of the 22 possible pathogens on the PCR panel, 12 (54.5%) were detected in this population. The most commonly detected pathogens were *Streptococcus pneumonia* (*n* = 83, 74.1%), enterovirus/rhinovirus (*n* = 69, 61.6%), *Moraxella cattarhalis* (*n* = 68, 60.7%), *Haemophilus influenza* (*n* = 54, 48.2%), and human parainfluenza virus 3 (*n* = 21, 18.8%). A median of three (IQR 2, 4) pathogens were detected per participant, with a range from zero to five. A median of two (IQR 1, 3) bacteria and one (IQR 0, 1) virus were detected per participant.

### 3.1. Latent Class Analysis

We performed latent class analyses and fit models with two through five subphenotypes to determine the optimal number of NPM subphenotypes in this population (Table 2). Using AIC, BIC, and number of participants per class, we determined the optimal fit of NPM subphenotypes was two classes. A less diverse subphenotype (*n* = 74) was identified and characterized by having fewer pathogens (median 2; IQR 1, 3) and specifically fewer bacteria (median 1; IQR 1, 2) (Table S2). A more diverse subphenotype (*n* = 38) was characterized by having more pathogens (median 4; IQR 3.25, 4.75) and specifically more bacteria with 34/38 participants in this class having three detectable bacteria and 4/38 participants having two detectable bacteria.

### 3.2. Linear Regression

Examination of the associations between NPM subphenotypes suggested that children with the more diverse subphenotype had no change in total airway resistance (R5Hz β = 0.03 cmH2O/(L/s), 95% CI −0.16, 0.21; *p* = 0.76) but an increase in large airway resistance (R20Hz β = 0.11 cmH2O/(L/s), 95% CI 0.02, 0.20; *p* = 0.02) and a decrease in small airway resistance (R5-20 β = −17.9%, 95% CI −35.6, −0.23; *p* = 0.047) compared with the less diverse phenotype following adjustment for child sex and height, maternal education, age at time of swab, and time between swab and lung function testing (Table 3). Sensitivity models additionally adjusting for parity, secondhand smoke exposure, number of people in the household, ethnicity, and child BMI did not substantively change these findings (Table S3).

We then explored associations between the four most commonly PCR-detected pathogens, specifically *Streptococcus pneumoniae*, enterovirus/rhinovirus, *Moraxella catarrhalis* and *Haemophilus influenzae*, each considered separately, and IOS variables (Table 4). These exploratory analyses suggest that presence of colonization with enterovirus/rhinovirus and *Moraxella catarrhalis* may be associated with a trend to decreased R5-20 (β = −16.1%, 95% CI −33.9, 1.7; *p* = 0.07 and β = −15.3%, 95% CI −32.1, 1.5; *p* = 0.07). Detection of enterovirus/rhinovirus was also associated with a trend to increased R20Hz (β = 0.08 cmH2O/(L/s), 95% CI −0.01, 0.18; *p* = 0.08). Detection of *Moraxella catarrhalis* was also associated with increased Fres (β = 3.63 1/s, 95% CI 0.46, 6.79; *p* = 0.03) and *Haemophilus influenzae* was associated with increased AX (β = 1.16 cmH2O/L, 95% CI 0.16, 2.16; *p* = 0.02).

## 4. Discussion

Impaired lung function and lung growth in childhood is a major risk factor for chronic respiratory disease over the life course. These analyses leverage a pregnancy cohort from rural sub-Saharan Africa with nasopharyngeal microbiota swabs from healthy infants and subsequent early childhood lung function as measured by impulse oscillometry and suggest that a less diverse respiratory microbiota in infancy is associated with increased small airway resistance in early childhood—a finding typically seen in asthma and other obstructive respiratory diseases. These results build on existing data from high-income countries and are a first step in elucidating mechanistic pathways that may increase risk for poorer lung health in a rural sub-Saharan population.

These data extend emerging evidence of the importance of respiratory microbiota on early life lung health, specifically lung function, and in a rural sub-Saharan African cohort relate microbiota carriage in infancy to future lung health. Once considered sterile, bacterial and viral colonization of the lungs has been demonstrated in children and infants [24,41]. In our study, targeted PCR of nasopharyngeal swabs in healthy infants found colonization with 12 unique bacteria and viruses. The four most commonly identified pathogens, *Streptococcus pneumoniae, Haemophilus influenzae*, enterovirus/rhinovirus, and *Moraxella catarrhalis*, were similar to those reported in the literature [14,15,19,20,24,42,43]. Other pathogens frequently discussed in studies, *Staphylococcus*, and *Cornebacteriacae* species, were not included in this PCR test and therefore their prevalence is unknown in our population. Respiratory syncytial virus and influenza A, which are frequently reported in other studies, were not found in this population, but given these pathogens frequently cause symptoms, it is not surprising they were not detected in our asymptomatic cohort. While this is not a comprehensive study of the microbiome, we did find polymicrobial colonization was present based on our PCR test, in the majority of infants in this cohort with a median of three (IQR 2, 4) pathogens present by age 4.3 months (IQR 2.9, 7.1).

These data suggest that lower NPM diversity in infancy is associated with increased small airway resistance. Increases in small airway resistance, as demonstrated by increased R5-20, are associated with asthma and increased risk of asthma exacerbations [44,45,46]. An increased AX, or total reactance, a measure of the change in the degree peripheral airway obstruction, has also been associated with asthma and an increased risk of losing asthma control in the subsequent 2–3 months [38,44,46]. A higher resonant frequency (Fres), the point at which the capacitive and inertive pressures in the lung equalize, is also associated with obstructive lung diseases, such as asthma, and restrictive lung diseases [44]. Overall, we find that a more diverse NPM subphenotype is associated with lower small airway resistance. Analyses of individual pathogens supported these findings. However, *Haemophilus influenzae* and *Moraxella catarrhalis* were also associated with increased AX and Fres, respectively. Continued follow up of lung health in this cohort is needed to understand the long-term lung health implications of these findings.

Studies from high-income countries support the importance of respiratory microbiota in the development of pulmonary disease. For example, respiratory microbiome colonization with *Streptococcus pneumonia*, *Haemophilus influenzae* and, in some studies, *Moraxella cattarhalis* has been associated with wheeze and asthma exacerbations [42,43]. Colonization in infancy is likely important. Bisgaard and colleagues demonstrated that neonatal colonization with these pathogens was associated with worse bronchodilator response at age five years, supporting our finding that respiratory microbiota can have a longstanding impact on lung function [24]. Our data suggests that a more diverse microbiota subphenotype in infancy was associated with lower resistance in the small airways, suggesting a possible protective effect. Interestingly, exploratory analyses of individual pathogens on child lung function showed trends but not similar effects. The bacteria and viruses that compose the microbiota do not exist in isolation and these analyses highlight the importance of employing analytic techniques that allow consideration of multiple, or the mixture of, microbes. A synergistic effect of microbes is likely important; it is possible that it is not a single pathogen, but rather the balance of respiratory microbiota, or overall diversity, that is essential.

Contrary to reports from high-income countries, the more diverse subphenotype identified in our cohort was characterized by the presence of *Streptococcus*, *Haemophilus* and *Moraxella* species—pathogens that in prior literature have been associated with wheeze and the development of asthma. Exploratory linear regressions demonstrated that enterovirus/rhinovirus and *Moraxella* species may drive this association. However, there are a number of differences between our population and those reported in prior literature which may contribute to these differences. The respiratory microbiome is shaped by many individual- and household-level factors, including mode of delivery, breastfeeding, number of siblings, and environmental exposures including air pollution and proximity to livestock to name a few [15,16,20,24,47,48]. For many of these variables, our population was homogeneous—for example, all households are agrarian with exposure to livestock, all children received pneumococcal vaccinations, and all children were breastfed through the first year of life. Given differences in our rural Ghanaian cohort and those cohorts reported from high-income countries, differences in microbiota and associations with respiratory phenotype are not surprising but do require confirmation and additional evaluation. Future work should also investigate whether these microbiota and lung function findings early in life will persist into adulthood.

Our study has several strengths. First, we leverage a prospective pregnancy cohort from rural sub-Saharan Africa, a population whose microbiota and respiratory phenotype has not previously been described. We assessed microbiota in healthy infants, prospectively collected data on important covariates, and employed a validated measure of lung function in early childhood. We applied a data-driven method to assess the microbiota mixture, allowing us to identify an underlying subphenotype as well as associations between these subphenotypes and future lung function. Our well-characterized cohort allowed for adjustment for a number of important covariates and a broad range of sensitivity analyses. As we continue to follow our cohort as they age, it will be important to repeat microbiota assessments and lung function to determine continued associations and whether microbiota in infancy can serve as a biomarker of future child respiratory health outcomes.

We also acknowledge limitations. We sampled the nasopharyngeal microbiota once in infancy, capturing one time point in a dynamic system. We limited our sample to asymptomatic infants at time of swab and without a pneumonia diagnosis within two weeks prior to or following swab to ensure a recent infection or course of antibiotics did not cause an acute change in the respiratory microbiota. However, it is possible that we were unable to detect clinically mild respiratory infections which could, in turn, transiently alter the microbiota. Additionally, the PCR analyses were limited to 22 common pathogens and did not provide a comprehensive assessment of all pathogens present in the nasopharyngeal microbiota or a full assessment of the microbiome. Other methods, including shotgun metagenomics, would provide a more complete picture of the nasopharyngeal microbiome and enable a better understanding of the diversity in our cohort. A larger sample size may have allowed us to detect additional subphenotypes and explore higher-level interactions, for example, sex-specific differences. Finally, our findings from a rural Ghanaian cohort may not be broadly generalizable.

## 5. Conclusions

These data from a rural Ghanaian cohort suggest an association between infant respiratory microbiota subphenotypes and future lung function, adding to the growing body of literature supporting the importance of the early life microbiome in future health. Further studies are required to replicate and extend these findings.

## Figures and Tables

**Table 1:** Participant characteristics (*n* = 112).

	Number	Percent
Child sex, Male	57	50.9
Secondhand smoke exposure, Yes	11	9.8
	**Median**	**IQR**
Height (m)	0.98	0.95, 1.01
Number of people in household	6	5, 8
Parity	3	1, 5
Age at nasal swab (months)	4.27	2.86, 7.11
Number of pathogens detected on PCR		
Bacteria	2	1, 3
Viruses	1	0, 1
Age 4 Impulse Oscillometry (IOS)		
R5Hz [cmH2O/(L/s)]	1.40	1.19, 1.65
R20Hz [cmH2O/(L/s)]	0.83	0.72, 0.97
R5-20 (%)	66.4	50.1, 96.9
X5 [cmH2O/(L/s)]	−0.32	−0.41, −0.20
Fres (1/s)	27.8	23.9, 34.5
AX (cmH2O/L)	5.60	4.37, 7.27
CO20Hz	0.94	0.90, 0.95
Time from nasal swab to IOS (Years)	3.57	3.37, 3.70

PCR = polymerase chain reaction.

**Table 2:** Fit statistics for latent class analysis demonstrating a two-class model as the best fit for this cohort.

Number of Classes	AIC	BIC	Entropy	Number of Individuals in Each Class				
				1	2	3	4	5
Nasal PCR								
Number of Classes								
1	873	905	3.7	112				
2	841	908	3.6	38	74			
3	878	982	3.6	44	52	16		
4	900	1039	3.6	42	27	41	2	
5	889	1061	3.5	11	25	32	42	2

AIC = Akaike information criterion. BIC = Bayesian information criterion.

**Table 3:** Associations between nasopharyngeal microbiota (NPM) subphenotypes and impulse oscillometry (IOS).

IOS Parameters	Unadjusted Model		Adjusted Model *	
	β (95% CI)	*p*	β (95% CI)	*p*
R5Hz [cmH2O/(L/s)]	0.02 (−0.16, 0.20)	0.82	0.03 (−0.16, 0.21)	0.76
X5Hz [cmH2O/(L/s)]	0.02 (−0.08, 0.11)	0.76	−0.01 (−0.10, 0.09)	0.91
R20Hz [cmH2O/(L/s)]	0.10 (0.01, 0.19)	0.03	0.11 (0.02, 0.20)	0.02
R5-R20 (%)	−18.1 (−34.9, −1.26)	0.04	−17.9 (−35.6, −0.23)	0.047
Fres (1/s)	1.48 (−1.86, 4.83)	0.38	1.32 (−2.10, 4.74)	0.45
AX (cmH2O/L)	0.57 (−0.44, 1.57)	0.27	0.65 (−0.39, 1.70)	0.22

* Model adjusted for child sex and height, maternal education; age at NPM PCR swab and time between NPM PCR swab and lung function testing. Beta interpreted as the difference in IOS variable between the more diverse subphenotype compared with to the less diverse subphenotype.

**Table 4:** Associations between specific nasopharyngeal pathogens and impulse oscillometry (IOS) *.

IOS Parameters	Streptococcus pneumoniae		Enterovirus/Rhinovirus	
	β (95% CI)	*p*	β (95% CI)	*p*
R5Hz [cmH2O/(L/s)]	−0.14 (−0.34, 0.06)	0.16	−0.02 (−0.21, 0.16)	0.83
X5Hz [cmH2O/(L/s)]	0.01 (−0.10, 0.11)	0.90	0.04 (−0.06, 0.13)	0.43
R20Hz [cmH2O/(L/s)]	−0.01 (−0.12, 0.09)	0.80	0.08 (−0.01, 0.18)	0.08
R5-R20 (%)	−11.5 (−31, 7.95)	0.24	−16.1 (−33.9, 1.7)	0.07
Fres (1/s)	0.11 (−3.62, 3.84)	0.95	1.76 (−1.67, 5.20)	0.31
AX (cmH2O/L)	0.21 (−0.94, 1.36)	0.72	0.06 (−0.99, 1.12)	0.91
	**Moraxella** **catarrhalis**		**Haemophilus** **influenzae**	
	β **(95% CI)**	***p***	β **(95% CI)**	***p***
R5Hz [cmH2O/(L/s)]	−0.02 (−0.19, 0.15)	0.81	0.02 (−0.16, 0.20)	0.84
X5Hz [cmH2O/(L/s)]	0.02 (−0.07, 0.11)	0.65	−0.07 (−0.16, 0.02)	0.12
R20Hz [cmH2O/(L/s)]	0.06 (−0.03, 0.15)	0.21	0.06 (−0.04, 0.15)	0.24
R5-R20 (%)	−15.3 (−32.1, 1.5)	0.07	−1.79 (−19.6, 16.0)	0.84
Fres (1/s)	3.63 (0.46, 6.79)	0.03	0.92 (−2.46, 4.30)	0.59
AX (cmH2O/L)	0.48 (−0.50, 1.46)	0.33	1.16 (0.16, 2.16)	0.02

* All models adjusted for child sex and height, maternal education, age at NPM swab, and time between NPM swab and lung function testing. Beta interpreted as the difference in the IOS variable between those with detected indicated pathogen compared with undetected.

## Data Availability

The de-identified data presented in this study is available on request from the corresponding author.

## Supplementary Materials

The following are available online at https://www.mdpi.com/article/10.3390/ijerph18147276/s1, Table S1: Bacterial and viral pathogens tested on PCR, Table S2: Detected rate of pathogens by PCR, Table S3: Sensitivity analysis: Associations between nasopharyngeal microbiota (NPM) subphenotypes and impulse oscillometry (IOS).
