# Supplementary material for: Infant Nasopharyngeal Microbiota Subphenotypes and Early Childhood Lung Function: Evidence from a Rural Ghanaian Pregnancy Cohort
Source: Int J Environ Res Public Health. 2021 Jul 7;18(14):7276. doi: 10.3390/ijerph18147276 (PMC8305530; doi:10.3390/ijerph18147276)
Supplement: Supplementary file 1 [file ijerph-18-07276-s001.zip › ijerph-1241687-supplementary.pdf]

Table S1. Bacterial and viral pathogens tested on PCR.

DNA agents	RNA agents
Chlamydophila pneumoniae	Influenza virus A
Legionella pneumophila	Influenza virus B
Mycoplasma pneumoniae	Respiratory syncytial virus A (RSV A)
Neisseria meningitidis	Respiratory syncytial virus B (RSV B)
Haemophilus influenzae	Human parainfluenza virus 1 (HPIV 1)
Streptococcus pneumoniae	Human parainfluenza virus 2 (HPIV 2)
Mycobacterium tuberculosis	Human parainfluenza virus 3 (HPIV 3)
Moraxella catarrhalis	Human parainfluenza virus 4 (HPIV 4)
Bordetella pertussis	Human metapneumovirus (MPV)
Adenovirus	Coronavirus OC43
	Coronavirus NL63
	Enterovirus/Rhinovirus

Table S2. Detected rate of pathogens by PCR.

Pathogen	Total (N=112)	Subphenotype 1 (N=38)	Subphenotype 2 (N=74)
Streptococcus pneumoniae	83 (74.1%)	38 (100%)	45 (60.8%)
Enterovirus/Rhinovirus	69 (61.6%)	25 (65.8%)	44 (59.5%)
Moraxella catarrhalis	68 (60.7%)	38 (100%)	30 (40.5%)
Haemophilus influenzae	54 (48.2%)	34 (89.5%)	20 (27.0%)
Human Parainfluenza virus 3 (HPIV 3)	21 (18.8%)	8 (21.1%)	13 (17.6%)
Human Parainfluenza virus 1 (HPIV 1)	4 (3.6%)	0 (0%)	4 (5.4%)
Coronavirus NL63	4 (3.6%)	4 (10.5%)	0 (0%)
Human Parainfluenza virus 2 (HPIV 2)	3 (2.7%)	3 (7.9%)	0 (0%)
Influenza virus A	3 (2.7%)	0 (0%)	3 (4.1%)
Coronavirus OC43	1 (0.9%)	1 (2.6%)	0 (0%)
Adenovirus	1 (0.9%)	0 (0%)	1 (1.4%)
Respiratory Syncytial virus A	1 (0.9%)	1 (2.6%)	0 (0%)

Table S3. Sensitivity analyses: Associations between Nasopharyngeal Microbiota (NPM) Subphenotypes and Impulse Oscillometry (IOS).

IOS Parameters	+ parity		+ second hand smoke exposure	
	**β (95% CI)**	**p**	**β (95% CI)**	**p**
R5Hz [cmH2O/(L/s)]	0.03 (-0.16, 0.21)	0.77	0.04 (-0.14, 0.22)	0.63
X5Hz [cmH2O/(L/s)]	-0.01 (-0.10, 0.09)	0.89	-0.01 (-0.10, 0.09)	0.86
R20Hz [cmH2O/(L/s)]	0.11 (0.02, 0.20)	0.02	0.11 (0.02, 0.21)	0.02
R5-R20 (%)	-17.87 (-35.63, -0.11)	0.049	-17.19 (-34.84, 0.46)	0.056
Fres (1/s)	1.32 (-2.12, 4.76)	0.49	1.16 (-2.25, 4.57)	0.50
AX (cmH2O/L)	0.65 (-0.40, 1.70)	0.22		
	**+ number of people in household**		**+ ethnicity**	
	**β (95% CI)**	**p**	**β (95% CI)**	**p**
R5Hz [cmH2O/(L/s)]	0.03 (-0.16, 0.21)	0.77	0.05 (-0.13, 0.24)	0.56
X5Hz [cmH2O/(L/s)]	-0.05 (-0.10, 0.09)	0.92	0.001 (-0.09, 0.10)	0.99
R20Hz [cmH2O/(L/s)]	0.11 (0.02, 0.20)	0.02	0.12 (0.03, 0.21)	0.01
R5-R20 (%)	-17.77 (-35.68, 0.14)	0.052	-17.23 (-35.18, 0.71)	0.059
Fres (1/s)	0.99 (-2.44, 4.42)	0.57	1.59 (-1.90, 5.08)	0.37
AX (cmH2O/L)	0.65 (-0.40, 1.71)	0.22	0.80 (-0.22, 1.83)	0.12
	**+ child BMI**			
	**β (95% CI)**	**p**		
R5Hz [cmH2O/(L/s)]	0.02 (-0.16, 0.21)	0.80		
X5Hz [cmH2O/(L/s)]	-0.01 (-0.10, 0.09)	0.91		
R20Hz [cmH2O/(L/s)]	0.10 (0.01, 0.19)	0.03		
R5-R20 (%)	-17.55 (-35.17, 0.28)	0.054		
Fres (1/s)	1.08 (-2.26, 4.42)	0.52		
AX (cmH2O/L)	0.61 (-0.43, 1.66)	0.25		

* Models adjusted for child sex, maternal education; age at NPM swab and time between NPM swab and lung function testing plus additional variables as indicated Beta interpreted as the difference in IOS variable between the more diverse subphenotype as compared to the less diverse subphenotype
